# Accidental digital epinephrine injection injury: a case of conservative management

**DOI:** 10.1093/jscr/rjab110

**Published:** 2021-04-19

**Authors:** Hatan Mortada, Bandari Abdullah Ahmed, Khalid Arab

**Affiliations:** Division of Plastic Surgery, Department of Surgery, King Saud University Medical City, King Saud University, Riyadh, Saudi Arabia; Plastic and Reconstructive Surgery Section, Department of Surgery, King Fahad Medical City, Riyadh, Saudi Arabia; Division of Plastic Surgery, Department of Surgery, College of Medicine, King Saud University, Riyadh, Saudi Arabia

## Abstract

The open access to epinephrine autoinjectors has resulted in an increased number of reports related to accidental injection into the digits. The appropriate management after accidental injection remains controversial. This study presents the first case in Saudi Arabia of a young man who accidentally injected epinephrine into the thumb and a literature review of the treatment options available. A 19-year-old man presented with accidental injection of 300 mcg of epinephrine into the volar pulp of his right thumb while treating an allergic reaction. The embedded needle was removed by countertraction and irrigation. The examination results were normal. The patient was discharged with prophylactic antibiotic and analgesia. Later, the puncture wound healed and vascularity and sensation remained intact. Conservative management and observation are advantageous in certain cases if vascular function is uncompromised. This case highlights the importance of education about the correct handling and administration of the epinephrine injection.

## INTRODUCTION

The rates of food- and drug-related allergies are increasing in the community [1]. The gold standard of emergency management of individuals at a risk of developing severe allergic reactions, such as anaphylaxis is the epinephrine injection [2]. The unrestricted access to epinephrine autoinjectors in the community has resulted in an increase in the number of reports related to accidental injection into the digits by patients, caregivers and healthcare professionals [3]. Approximately 1 in every 50 000 attempts at administration results in an accident [4, 5]. The mechanism of action of epinephrine can result in vasoconstriction by stimulation of the alpha one and alpha two receptors on the vascular smooth muscles, which, if injected into the digit, can lead to vascular compromise and severe ischemic necrosis [6]. Despite the increasing number of reports of accidental injection of epinephrine into the digits, the most appropriate form of management remains controversial. Consequently, we are unaware of any previously published case reports in Saudi Arabia managed explicitly by conservative treatment after the accidental injection of epinephrine. Thus, in this study, we present the first case in Saudi Arabia of a young man who accidentally injected epinephrine into the thumb while handling an autoinjector device and was treated with a conservative approach.

## CASE PRESENTATION

**Figure 1:**
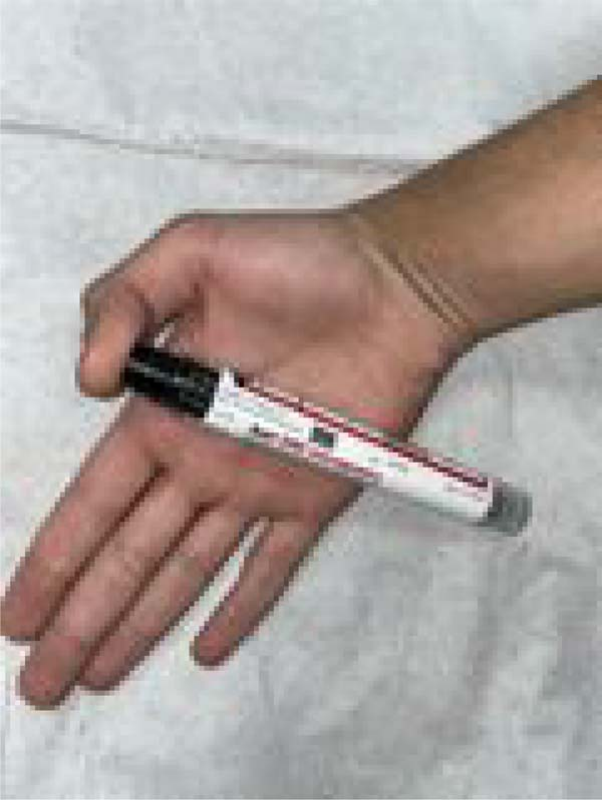
Appearance of the right thumb at presentation.

A 19-year-old man presented to the emergency department (ED) with pain and discomfort after accidentally injecting himself with the epinephrine autoinjector while attempting to treat an allergic reaction in a family member (Fig. 1).

**Figure 2:**
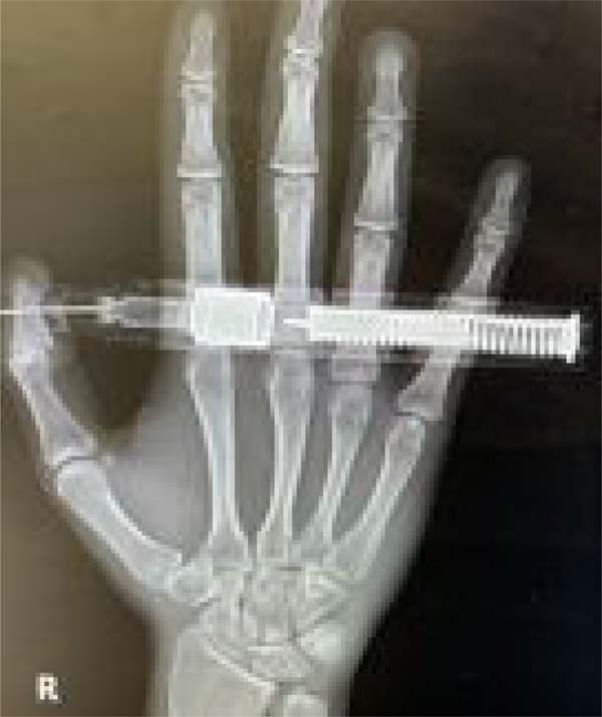
Posteroanterior view of the embedded needle on the right distal phalanx of thumb.

On presentation, there was no numbness or paresthesia. The accident occurred 30 min before his arrival to the ED. He accidentally injected 300 mcg of epinephrine (0.3 mg/0.3 ml, and 1:1000) into the volar pulp of his right thumb using the autoinjector. Initially, he experienced moderate pain in the right thumb, which subsided upon arrival to the ED. On examination, a puncture wound was visible on the pulp of the right thumb, distal to the interphalangeal joint. The injector needle was firmly embedded into the volar aspect of the distal phalanx of the right thumb. The digit was warm and pink in color, and the capillary refill was less than 2 s. Sensation was retained in the area distal to the puncture site. The active and passive range of motion of the thumb were intact. His vital signs and oxygen saturation were normal, and there were no signs of impaired peripheral perfusion. A hand radiography did not show a fracture of the distal phalanx of the thumb or the presence of foreign bodies (Fig. 2).

**Figure 3:**
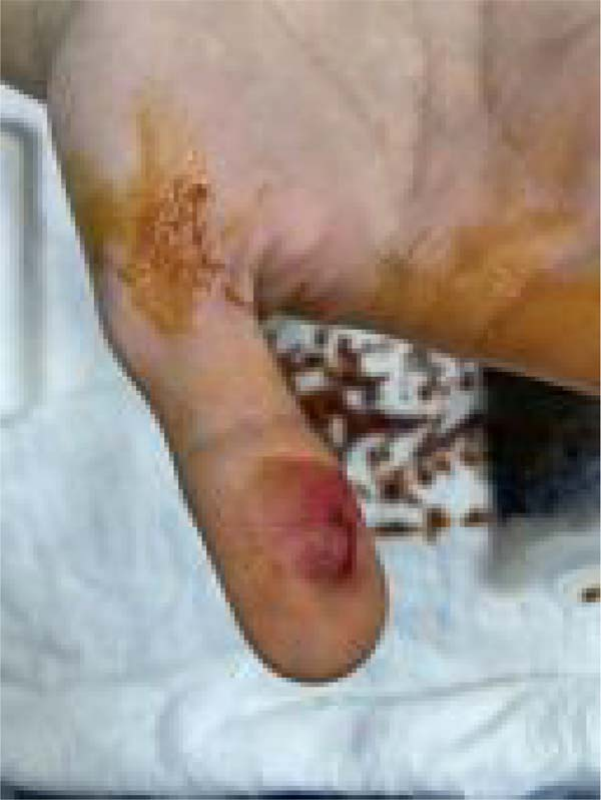
After needle removal under sterile condition.

We successfully removed the embedded needle in a sterile manner through moderate countertraction (Fig. 3) and irrigation with 2 l of normal saline with povidone. The X-ray was repeated after removal to rule out the presence of any residual foreign bodies. The examination was repeated and the results showed a normal and intact thumb.

**Figure 4:**
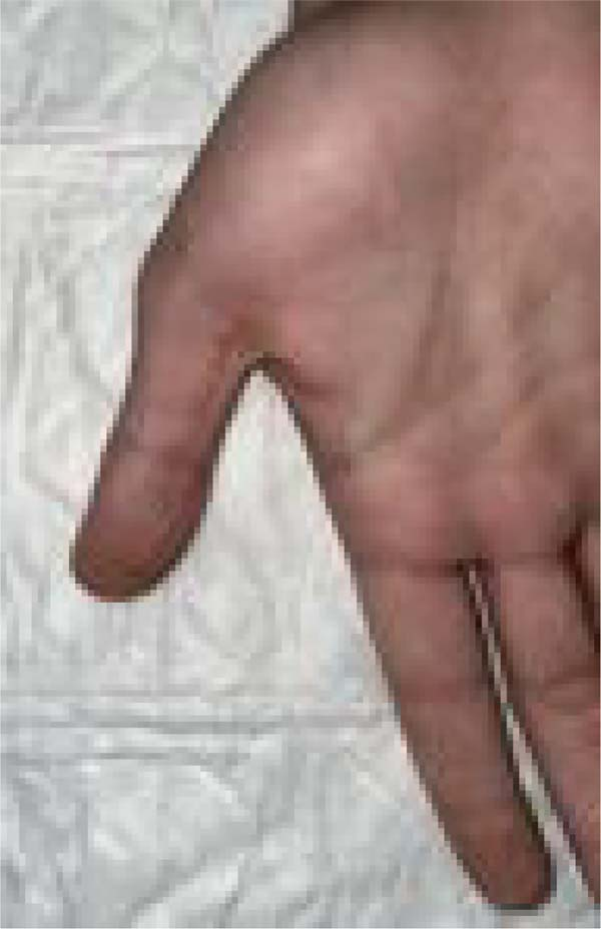
On follow-up, 7 days after the accident. The wound had healed completely.

The patient remained under observation for a couple of hours. He was discharged after his condition remained stable with normal vascular findings. He was instructed to visit the ED if his condition worsened or if any signs of vascular compromise occurred. He was also educated on the proper handling and administration of the epinephrine autoinjector. He was discharged on prophylactic antibiotic and analgesia. On his follow-up visit 7 days after the accident, the puncture would have healed and vascularity and sensation were intact (Fig. 4).

## DISCUSSION

The mechanism of action of epinephrine in anaphylactic reactions includes stimulation of vascular resistance and blood pressure, resulting in bronchodilation [7, 8]. Epinephrine when used with local anesthesia also acts as a vasoconstrictive agent to minimize bleeding. The use of epinephrine in end artery structures remains controversial due to the risk of vascular compromise, necrosis and loss of digits. Recently published studies on the use of lidocaine and epinephrine have reported adverse consequences [9, 10]. Denkler *et al.* reported 48 cases of finger necrosis after anesthetic blocks [11]. However, there are few reports on the accidental injection of epinephrine. The first report of accidental epinephrine injection into the finger was published in 1989 [12]. Since then, many different management options have been reported, including conservative management and subsequent spontaneous resolution [12]. The main issue in choosing the appropriate treatment strategy is that the quantity of epinephrine injected into the digit is impossible to determine and as such an incorrect antidote dose can worsen the vascularity due to the pressure effect. This leads to digital ischemia within the first hour of injection. It has been proposed that a watchful waiting approach is needed upon presentation if the patient has no signs or symptoms of vascular compromise, and all other findings are normal [13]. This strategy is in agreement with our management of the reported case. When choosing a management approach, ruling out digital necrosis should be the first consideration. Nevertheless, patients treated with the pharmacological intervention had a longer time in the ED, suffered from extreme reperfusion pain and had a longer recovery time [5]. An analysis of 59 cases of digital high-dose epinephrine injection from 1989 to 2005 was published in 2017 by Fitcharles-Bowe *et al.* [5]. In this study, 32 patients underwent a watchful waiting management plan, 25 patients received pharmacological intervention and the management was unknown in two patients. The most commonly used pharmacological agent was phentolamine (60%). Although none of the patients suffered from digital necrosis, those managed with phentolamine experienced quicker symptom resolution and perfusion normalization. Digital ischemia and the outcomes of the accidental injection of epinephrine can be influenced by the patient's age, past medical history, the dosage of epinephrine and the exact digital injection site. Therefore, the appropriate management plan will depend on this data. The rarity of this accident implies that treatment options remain controversial.

In this case report, we outlined the presentation and management options for accidental digital epinephrine injection, highlighting the advantages of conservative management and observation. However, pharmacological intervention is needed if necrosis and vascular compromise signs are present.

## CONFLICT OF INTEREST STATEMENT

None declared.

## FUNDING

None.
